# The Effect of EFL Teacher Apprehension and Teacher Burnout on Learners’ Academic Achievement

**DOI:** 10.3389/fpsyg.2021.839452

**Published:** 2022-01-20

**Authors:** Zhiping Wang

**Affiliations:** Foreign Languages School, Nanyang Normal University, Nanyang, China

**Keywords:** academic achievement, teacher burnout, teacher apprehension, foreign language learning, EFL teachers

## Abstract

This review aims at investigating the related studies on the role of teacher burnout and teacher apprehension in learners’ academic achievement. The negative effect of teacher burnout on learners’ academic achievement has been corroborated in the review of the literature. Furthermore, the effect of teacher apprehension on learners’ academic success has not been widely studied. However, some stressors such as having insufficient L2 knowledge, cultural differences, and classroom management problems can affect learners’ academic achievements. Finally, the pedagogical implications are illuminated for teachers, administrators, researchers, managers, teacher trainers, and counselors to decrease teacher burnout and apprehension and to develop language teaching quality in the language educational system. Some suggestions for further research are also provided to expand the current literature on teacher burnout and apprehension in English as a Foreign Language (EFL) contexts.

## Introduction

Instruction is considered a job full of apprehension, stress, and anxiety which affect teachers and their quality in language contexts (Montgomery and Rupp, 2005). Apprehension can influence teachers’ psychological well-being and this can decline teachers’ performance in a classroom context (Folkman et al., 1986). Apprehended teachers do not have an appropriate relationship with learners. Therefore, learners’ engagement and enjoyment are affected by teacher apprehension (Wentzel, 2010). Continuous apprehension and stress of teachers lead to teacher burnout which may affect their performance negatively (Sapolsky, 1998). Sapolsky (1998) argued that teacher burnout happens when a teacher negatively appraises the difficulties of his occupation and his incapability to encounter that difficulty. The transactional model of stress suggests that stress and burnout happen simultaneously (Belcastro and Gold, 1983). Recently, teacher quality has been a controversial issue in teacher education, and numerous educational practitioners attempted to apply educational theories in English as a Foreign Language (EFL) contexts. Nevertheless, foreign language teachers, who are responsible for increasing EFL learners’ engagement and their capability, have not been considered noticeably in educational contexts (Brown and Lee, 2015). Therefore, it is required to consider various features which can negatively affect teacher quality in L2 contexts (Fathi and Derakhshan, 2019). Teacher apprehension and burnout are likely to impede teacher’s endeavors to apply effective methodologies which may affect learners This review tends to consider the effects of teacher apprehension and burnout, as negative emotions, on EFL learners’ success in academic contexts.

## Literature Review

### Teacher Burnout

According to Maslach (1976), burnout is defined as “a syndrome of emotional exhaustion, lack of personal accomplishments, and depersonalization and the fire of enthusiasm and commitment to success being reduced to ashes” (p. 1). Teaching is regarded as one of the occupations which is assumed to have high levels of burnout (Hakanen et al., 2006). Bakker and Costa (2014) asserted that emotional exhaustion occurs in teachers who are mentally tired. They also maintained that depersonalization in teachers leads to the adoption of a negative approach in coping with events. Yildiz (2015) argued that depersonalization is an emotional disconnection that disturbs both teachers’ individual and occupational positions. Decreased individual achievement is also defined as a failure in occupational capability and efficiency (Kim et al., 2019). Chang (2009) classified the causes of burnout into personal (e.g., gender, age, job experience, etc.), administrative (e.g., insufficient income, classroom size, the socioeconomic position of the organization, workload, etc.), and interactional (student-teacher, teacher-teacher, and teacher-principal interaction). Some investigations have been done on the reasons for teacher burnout. Chang (2013) found out that personal characteristics do not significantly predict teacher burnout. However, Foley and Murphy’s (2015) study revealed that Irish teachers’ individual differences, working contexts, and classroom management are significantly related to burnout. Kara (2019) found a significant correlation between individualized variables such as gender, years of experience, marital status, and burnout. Moreover, it is revealed that there is a negative correlation between job satisfaction and teacher burnout. Herman et al. (2018) argued that instructors who participate in social events have lower levels of burnout. Mahmoodi and Ghaslani (2014) found a negative correlation between burnout and emotional intelligence. It means that instructors with high levels of positive emotions are less likely to experience burnout. Capone et al. (2019) also argued that burnout is regarded as a vital component in the mental well-being of instructors as it plays a mediating role between work variables and depression.

### Teacher Apprehension

Kyriacou (2001) defined apprehension as the future–based anxiety and stress which occur in unpleasant special contexts. He mentioned that anxious individuals usually have some problems in communicating with people. According to Horwitz et al. (1986), apprehension, as a subcomponent of anxiety is related to stimulation of the autonomic nervous system. In the teaching context, internal and external stressors are experienced by teachers. Being incompetent in communication, feeling loneliness, and lacking self-esteem are examples of internal stressors. On the other hand, having deficient knowledge about recent instructional materials, inefficient rapport with learners, time pressure during instruction, and struggling with demotivated learners can be the instances for the external stressors (Kyriacou, 2001). Communication apprehension is a common type of teacher apprehension that is highly related to nervousness about interacting with learners (Kim and Kim, 2004). In a foreign language learning context, Horwitz et al. (1986) defined communication apprehension as “a distinct complex of self-perceptions, beliefs, feelings, and behavior related to classroom language learning arising from the uniqueness of the language-learning process” (p. 28). Communication apprehension has been still widely studied in recent years in communication research (Bragg, 2017). Since communication apprehension is a fundamental issue that restrains the received comprehensible input, it plays a key role in specifying achievement in language learning (Darmawangsa et al., 2020). Numerous studies have been done to investigate teachers’ source of apprehension. Goldast et al. (2021) indicated some teachers’ lack of self-esteem, L2-related problems, cultural differences, classroom management, and organizational problems are significantly related to teacher apprehension. In a study, Ghanizadeh et al. (2020) found out that instructors’ identity and self-esteem are significantly correlated with teacher apprehension. Shillingford-Butle et al. (2012) also argued that teacher-parent interactions, instructive policies, and struggling with other instructors can lead to teacher apprehension.

### The Effect of Teacher Burnout on Learners’ Academic Achievements

So far, many studies have been done on the emotional features (e.g., well-being motivation, personality, etc.), which may be considered as crucial factors in achievement in the language learning context (Madigan and Curran, 2021). Teacher burnout, as a specific component of teacher well-being, has significant effects on learners’ academic performance (Schleicher, 2018). Brouwers and Tomic (2000) argued that teacher burnout leads to poor classroom management in dealing with learners’ troublesome classroom behaviors. They also asserted that teacher burnout can be a reason for poor academic performance. Moreover, Klusmann et al. (2008) mentioned that teacher burnout, described by disengagement and low levels of resilience, results in EFL learners’ poor performance in language learning. In a study, Madigan and Kim (2020) found out that teacher burnout is significantly correlated with poorer educational success and less motivation. However, they did not find any correlation between teacher burnout and learners’ well-being. Arens and Morin’s (2016) study revealed a significant negative correlation between teacher burnout and learners’ cognitive and non-cognitive academic results. Atik and Çelik (2021) also found out that academic engagement and motivation are correlated with each other. However, teacher burnout acts as a mediator role between them. Shen et al. (2015) argued that teachers, who suffer from burnout, have negative attitudes toward themselves which may affect their performance and learners’ academic achievements. Overall, the above-mentioned studies showed that teacher burnout provides less encouraging language teaching context to the learners, which can bring about low levels of academic achievement among them.

### The Effect of Teacher Apprehension on Learners’ Academic Achievement

In earlier investigations (e.g., Botes et al., 2020; Agrawal and Krishna, 2021; Jahedizadeh and Ghanizadeh, 2021), the role of learner apprehension in their academic engagement, achievement, and the sources of achievements have been highlighted. However, instructors, especially those with a lack of outgoing character, have key roles in learners’ participation in class (Li, 2021). Zhang (2009), in his study, found out that teacher apprehension and anxiety induce unbalanced conditions in the workplace, reduce productivity, and increase the rate of their mistakes. Moreover, his study revealed that emotional stress disturbs the information-transmission instruction approach. Yazhuan et al. (2010) argued that teacher apprehension influences their job performance and causes a significant gap between themselves and learners and it decreases personal accomplishments. Awan et al. (2010) argued that language anxiety can negatively affect learners’ achievement. They pointed out that classroom context should be more positive and appealing. They maintained that teachers should meticulously cope with apprehension-provoking states. Yildirim’ 2012 study revealed that teacher support, their well-being, and motivation can significantly affect learners’ academic achievement. Herman et al. (2018) found out that teachers’ higher levels of apprehension, burnout and their lower levels of self-efficacy were significantly correlated with learners’ poor performance in language contexts. Ramberg et al. (2019) found a negative correlation between teacher apprehension, exhaustion, sadness, and learners’ school satisfaction and academic engagement. Li (2021) also argued that instructors’ apprehension has a significant effect on learners’ engagement in foreign language tasks and this results in poor academic performance. Skinner (2016) pointed out that learners’ disengagement is correlated with teachers’ extreme conduct including indifference, abhorrence, resignation, and decreased attempts and anxiety. Greenier et al. (2021) also identified the harmful effects of teacher apprehension on learner engagement since teacher apprehension can avoid learners’ opportunities to engage and express themselves in classroom contexts. Xie and Derakhshan (2021) argued that learner disengagement in learning contexts occurs when teachers have a higher level of anxiety and lower levels of rapport with learners.

## Implications and Suggestions

This review aimed at investigating the role of teacher burnout and teacher apprehension in the learners’ academic achievement. The review improved our knowledge about teacher burnout and apprehension, and their effect on learners’ academic achievement. The related literature has shown a negative effect of teacher burnout and their apprehension on the learners’ academic achievement. Considering the related studies on the relationship among affective factors, it can be mentioned that teachers should be assisted to control, adjust, and regulate their feelings in language teaching contexts. This review implies that instructors can change learners’ academic engagement and achievement both by using different approaches and controlling their outward feelings. The review has some possible implications to support instructors in enhancing learners’ achievement. The administrators should ameliorate EFL contexts in support of instructors to develop their scope of decision making in opting out materials, teaching methodology, and to respect their occupational status. Administrators are required to organize a learning context that reduces teacher burnout, particularly their depersonalization, and increases their achievement. Since decreased learner achievement is the most pre-dominant aspect of teacher burnout, the provision of strategies for burnout avoidance in pre-service and in-service teacher training programs can be helpful. Moreover, teacher educators are required to prepare some courses for teachers. These courses should highlight instructors’ insights about their efficiency in the improvement of learners’ academic achievements. They can train teachers to change their attitudes and raise their awareness about the internal and external burnout components, and develop their objectives along with their individualized achievement. Therefore, apprehension and burnout control is a deep-rooted intervention in contributing teachers to learn how to deal with their moods and regular stressors (Herman and Reinke, 2015). Lack of regulating or controlling teachers’ affections may diminish the enjoyment of learners which may trigger teacher educators to consider this issue in practical aspects (Schutz and Zembylas, 2009). The managers of private language institutes should be acquainted with the main stressors influencing their EFL instructors. They can decrease educational workloads by meeting the needs of teachers in EFL classrooms, identifying teachers’ accomplishments in order to develop instructional efficiency, and concentrating on the growth of instructors’ self-confidence and classroom management strategies. In order to reduce the stress, they should be wary about the changes in organizational aspects by supporting job security and providing acceptable services. Likewise, school managers are recommended to develop educational workshops considering teacher apprehension, teacher burnout, and their effects on learners’ academic knowledge. They can also provide lectures about the way teachers can reduce external stressors such as time pressure and communication anxiety to amplify their ability for defying teacher burnout (Carson et al., 2011). Finally, the significance of teacher burnout and apprehension allows consultants to develop programs to reduce the effect of these variables on learning performance. They can identify teachers who need some support by arranging them based on their anxiety and burnout levels.

This review has some suggestions for further research. Most importantly, some investigations should be done to distinguish between instructors’ apprehension and stress, while they might seem identical in the instructive environments. It is also important to investigate the effect of burnout on instructors’ private lives. Moreover, learners’ family support and its effect on internal and external teacher burnout should be examined for the future. Future investigations can also compare various scales of measuring teacher apprehension and burnout. Many investigations on burnout should be done in different countries with various geographical, national, and cultural contexts. A longitudinal study can also be done to follow up the effect of EFL teacher burnout and his apprehension on EFL learners’ academic engagement, grittiness, and their foreign language enjoyment. Some investigations should also be carried out on the relationship between teacher burnout and mindset. Studies should be done on the effect of video games for decreasing learner and teacher burnout and teacher communication apprehension. The role of teacher apprehension and burnout in learners’ well-being needs to be studied. Learners’ performances in EFL contexts may bring about teacher burnout (Hoglund et al., 2015), which signifies probable mutual relations that would need to be considered for the future. Moreover, future research can highlight the role of gender, instructional experience, educational level in EFL teacher burnout and teacher apprehension. Furthermore, the relationship between EFL teachers’ emotional intelligence and their burnout and apprehension in foreign language learning backgrounds can be examined for the future. Studies should be done on the effects of EFL teachers’ and learners’ communication apprehension and their working memory. Besides, the relationships between EFL teacher self-efficacy, burnout, and apprehension need to be examined. The effect of empowering teachers on the improvement of language skills should be studied in detail. Also, the reciprocity of the relationship between EFL teacher apprehension and burnout should be scrutinized in the future. Future studies may entail the examination of other variables such as EFL instructors’ extroversion, introversion, and their relation with burnout and apprehension. Finally, since online language learning has revolutionized language teaching approaches during the COVID-19 pandemic (Wang and Derakhshan, 2021), the effects of teaching contexts (in class, online, and hybrid) on teacher burnout and teacher apprehension are required to be examined.

## Author Contributions

The author confirms being the sole contributor of this work and has approved it for publication.

## Conflict of Interest

The author declares that the research was conducted in the absence of any commercial or financial relationships that could be construed as a potential conflict of interest.

## Publisher’s Note

All claims expressed in this article are solely those of the authors and do not necessarily represent those of their affiliated organizations, or those of the publisher, the editors and the reviewers. Any product that may be evaluated in this article, or claim that may be made by its manufacturer, is not guaranteed or endorsed by the publisher.
